# Supplementary material for: ERp29 Attenuates Nicotine-Induced Endoplasmic Reticulum Stress and Inhibits Choroidal Neovascularization
Source: Int J Mol Sci. 2023 Oct 24;24(21):15523. doi: 10.3390/ijms242115523 (PMC10649101; doi:10.3390/ijms242115523)
Supplement: Supplementary file 1 [file ijms-24-15523-s001.zip › ijms-2653565-supplementary.pdf]

Supplementary Table S1

Target	Source	Vendor
GRP78 (3177)	Rabbit	Cell Signaling Technology, MA, USA
CHOP (L63F7)	Rabbit	Cell Signaling Technology, MA, USA
β-actin (13E5)	Rabbit	Cell Signaling Technology, MA, USA
ERp29(37555)	Rabbit	Signalway Antibody LLC, Greenbelt, USA

Supplementary Table S2

ERP29 FORWARD	5'-ACCCACGATTCTGAGCCCTGAG-3'
ERP29 REVERSE	5'-CCCAGACCAGCTAGAGACATCCC-3'
CD11b FORWARD	5'-GTTGTGACTTATGACCTGGCTCTGG-3'
CD11b REVERSE	5'-TGTCTGTCTGCGTGTGCTGTTC-3'
CD14 FORWARD	5'-AGCCTAGACCTCAGCCACAACTC-3'
CD14 REVERSE	5'-CAGCCCAGCGAACGACAGATTG-3'
CD163 FORWARD	5'-ATGCTTCCATCCAGTGCCTC-3'
CD163 REVERSE	5'-CACAAACCAAGAGTGCCGTGA-3'
CD206 FORWARD	5'-GTTCACCTGGAGTGATGGTTCTC-3'
CD206 REVERSE	5'-AGGACATGCCAGGGTCACCTTT-3'
IL-10 FORWARD	5'-GCCGTGGAGCAGGTGAAGAATG-3'
IL-10 REVERSE	5'-ATAGAGTCGCCACCCTGATGTCTC-3'
CCL-17 FORWARD	5'-CCCAACAACAAGAGAGTGAAGAATGC-3'
CCL-17 REVERSE	5'-CAACGGTGGAGGTCCCAGGTAG-3'
iNOS FORWARD	5'-CGGCAAACATGACTTCAGGC-3'
iNOS REVERSE	5'-GCACATCAAAGCGGCCATAG-3'
CD80 FORWARD	5'-ATAACAGTGTCCGCAGAAGCAAGG-3'
CD80 REVERSE	5'-CGTAAAGGGCAAGGTGGGGTAATC-3'
CCR7 FORWARD	5'-GCTGTGGTCGTGGTCTTCATAGTC-3'
CCR7 REVERSE	5'-AGGCGATGTTGAGTTGCTTACTGAG-3'
